# Household Water Chlorination Reduces Incidence of Diarrhea among Under-Five Children in Rural Ethiopia: A Cluster Randomized Controlled Trial

**DOI:** 10.1371/journal.pone.0077887

**Published:** 2013-10-23

**Authors:** Bezatu Mengistie, Yemane Berhane, Alemayehu Worku

**Affiliations:** 1 College of Health Sciences, Haramaya University, Harar, Ethiopia; 2 Addis Continental Institute of Public Health, Addis Ababa, Ethiopia; 3 School of Public Health, Addis Ababa University, Addis Ababa, Ethiopia; Aga Khan University, Pakistan

## Abstract

**Background:**

Household water treatment has been advocated as a means of decreasing the burden of diarrheal diseases among young children in areas where piped and treated water is not available. However, its effect size, the target population that benefit from the intervention, and its acceptability especially in rural population is yet to be determined. The objective of the study was to assess the effectiveness of household water chlorination in reducing incidence of diarrhea among children under-five years of age.

**Method:**

A cluster randomized community trial was conducted in 36 rural neighborhoods of Eastern Ethiopia. Households with at least one child under-five years of age were included in the study. The study compared diarrhea incidence among children who received sodium hypochlorite (liquid bleach) for household water treatment and children who did not receive the water treatment. Generalized Estimation Equation model was used to compute adjusted incidence rate ratio and the corresponding 95% confidence interval.

**Result:**

In this study, the incidence of diarrhea was 4.5 episodes/100 person week observations in the intervention arm compared to 10.4 episodes/100 person week observations in the control arm. A statistically significant reduction in incidence of diarrhea was observed in the intervention group compared to the control (Adjusted IRR = 0.42, 95% CI 0.36–0.48).

**Conclusion:**

Expanding access to household water chlorination can help to substantially reduce child morbidity and achieve millennium development goal until reliable access to safe water is achieved.

**Trial Registration:**

ClinicalTrials.gov NCT01376440

## Introduction

Diarrheal disease kills 1.5 million people mostly children under the age of five years in developing countries each year [1]. Many of infectious agents causing diarrhea are potentially water borne transmitted through contaminated water [2]. Even though Millennium Development Goal (MDG) of drinking water is achieved, 780 million people lack access to improved water sources and 2.5 billion lack improved sanitation worldwide, rural population are disproportionately undeserved [3]. Even water from improved source is not always safe [4]. Furthermore, water collected from initially acceptable microbial quality, it often becomes contaminated with pathogens during transport and storage [5].

To overcome the difficulties in providing safe water, point-of-use water treatment has been advocated as a means to improve access to potable water and decrease the global burden of diarrheal diseases [2], [6], [7]. However, the effect of household water disinfection with chlorine on diarrhea episode reduction is variable, ranging from no protective effect to 85 percent reduction [8]–[16]. The studies on effectiveness of water quality interventions in reducing diarrhea have been flawed due to responder observer biases [2], [17]. Uptake and use is low among rural population who are more at risk of water borne disease [18]. It is difficult to identify the population that benefit most from the potential effect of the intervention [17].

Thus, the main aim of this intervention study was to determine the effectiveness of household water chlorination (point-of-use water treatment) in reducing diarrhea incidence among children under-five years of age in rural community of Eastern Ethiopia.

## Methods

### Ethics Statement

The study was reviewed and approved by the Haramaya University, College of Health Science Ethical Review Committee. Consent was obtained from district administration, district health department, community leaders. Written consent was also obtained from the primary caregivers of children. Field workers provided Oral Rehydration Solution (ORS) obtained from Kersa district health department for the children with diarrhea and advised their caregiver to take them to the nearby health facility for further treatment. Control communities received the intervention after the completion of the study. The protocol for this trial (Protocol S1) and supporting CONSORT checklist (Checklist S1) are available as supporting information.

### Study Setting

The study was conducted in rural Kersa Demographic and Health Research Centre (KD-HRC) Field Site, Kersa district, Eastern Ethiopia. It is located in kersa district which is about 482 kms far from the capital, Adis Ababa**.** According to the 2007 baseline survey, the study area has a population of 47,036 (8960 households) distributed in 10 kebeles (smallest administrative unit with a population of 5,000) of which 7870 are children under-five years of age. All the households had no running water. Families collect water from springs, streams/rivers, or wells and store in 20 liter jerry-can.

### Study Design and Procedure

We conducted randomized controlled, parallel group, field trial to assess the effectiveness of household chlorination in reducing diarrhea episode. The study site was selected by Haramaya University in 2007 to serve as demographic surveillance and health research center. It is one of the six Demographic Surveillance Sites (DSS) in the country. Ethiopian statistics authority has demarcated population enumeration villages (clusters) for 2007 Ethiopian population census across the country. Clusters are distinct neighborhoods with defined geographical boundaries. Rural KDS-HRC has 64 clusters and all were eligible for the study. Ethiopian central statistics authority statistician randomly selected 36 clusters from district population enumeration areas using computer generated random sampling. Field workers conducted census identified households in the selected clusters that had at least one child under-five years of age. Twenty four children (median of 16 households) were selected in each cluster by using simple random sampling from the list of households that contain the number of children in the presence of community leaders and some residents for follow up.

**Figure 1:**
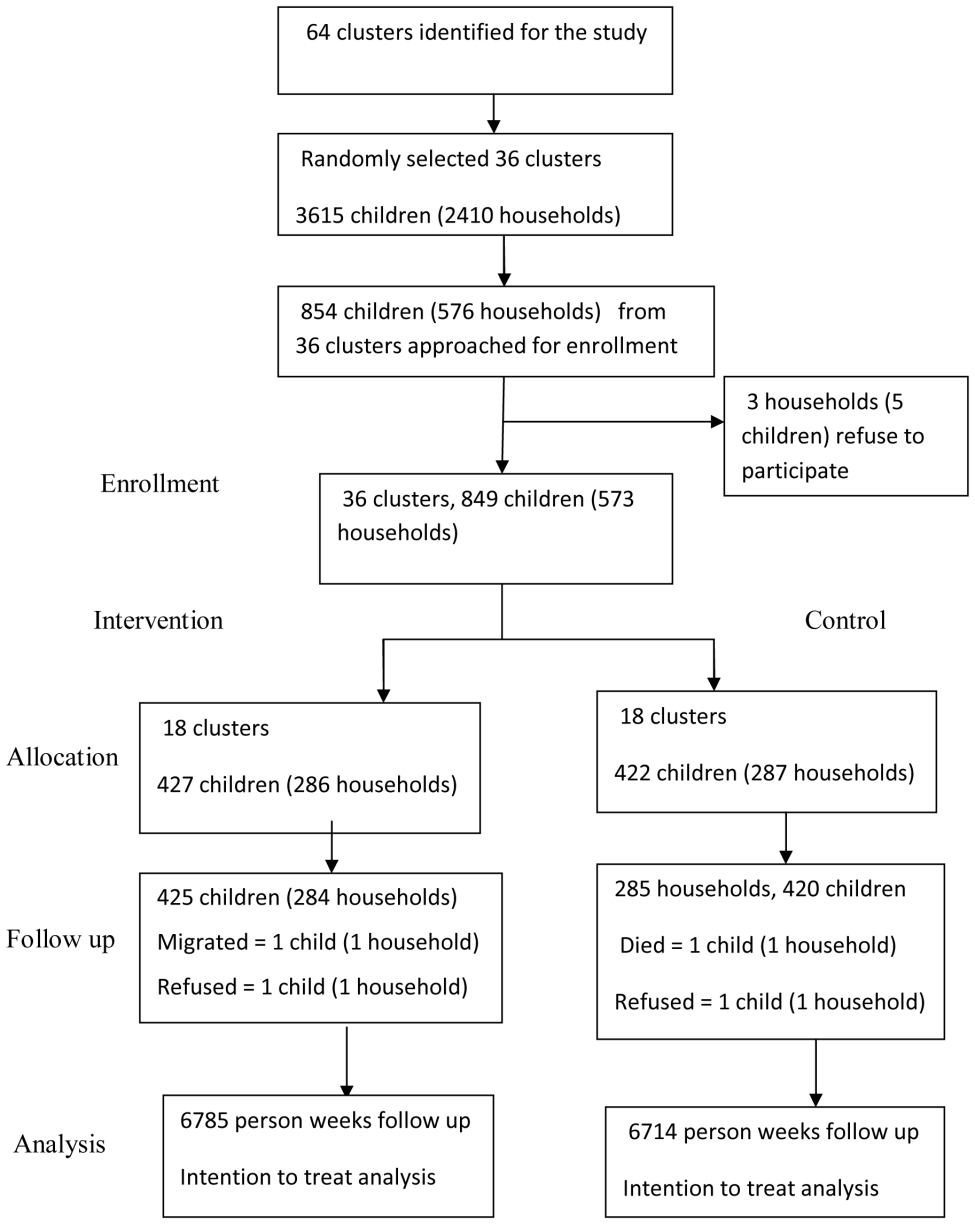
Community randomized trial flow of participants on household water chlorination, eastern Ethiopia, 2011.

The randomization of clusters was done in a meeting with community leaders and representative from the health department. Each cluster code was written on a separate paper in front of the community leaders and put in a box. They agreed the first 18 draws to be assigned in the intervention arm and the remaining in the control. One of the community leaders draw 18 clusters consecutively from the box and assigned in the intervention arm. The remaining 18 clusters assigned in the control group. Field workers approached residents of selected households and completed baseline survey. Sodium hypochlorite (intervention) was distributed for all the households in the intervention arm. We used cluster level randomization to avoid ethical concerns and minimize the potential transfer of the intervention between the two groups (figure 1).

### Sample Size

The sample size was calculated using methods published by Hayes and Bennett [19], assuming 11% incidence of diarrhea among children in the control group based on previous study [20], 80% power, 10% drop out, 95% confidence interval and design effect of three from clustering. Accordingly, we aimed to enroll 18 clusters per arm with 24 children under the age of five years per cluster followed for 16 weeks (which would provide 6921 person weeks of observation in each group) to get sufficient power to detect 40% reduction in the incidence of diarrhea in the intervention group among children under-five years of age.

### Intervention

The intervention for this study was 1.25% sodium hypochlorite branded locally known as “WaterGaurd” and obtained from population service international (PSI) manufactured specifically for home water disinfection. Local women were selected to distribute sodium hypochlorite for the intervention households with no charge to treat their stored water at home for 16-weeks from June to October 2011. They also explained how to treat water with chlorine and demonstrated how to use the disinfectant. Control groups continued their usual practice with respect to drinking water. Both intervention and control groups collect and store water with 20 liter jerry cans. In many African countries 20 liter jerry cans are used to transport and store water at home and are good options for safe storage [21].

### Data Collection

A baseline survey was conducted on the demographic and socioeconomic condition, sources of water, access to and quality of water, water handling practice, sanitation, hygiene and pre-intervention diarrhea rates. The questionnaire was translated from English to the local language, Oromifa, back translated in to English and administered to the mother/caregiver by Local language.

The primary outcome was the occurrence of diarrhea among children under-five years of age. Diarrhea is defined as three or more loose or watery stools in 24 hours or more frequently than normal for an individual [1]. We defined a new episode of diarrhea if it occurred after a period of three diarrhea free days [22], [23]. We calculated the incidence of diarrhea as the number of new episodes divided by the total number of person–weeks observation [24]. The survey instrument was pre-tested in the nearby villages and amended based on the comments from the pretest. Field workers obtained data on the occurrence of diarrhea, water treatment practices and residual chlorine on weekly bases during the study.

The secondary outcome was compliance of the intervention. It was assessed on two unannounced and regular weekly visits using free residual chlorine measured with residual chlorine test kit (Wagetech 225 comparator color disc). It is a color wheel test kit that uses DPD (N,N diethyl-p-phenylene diamine) tablet.

Water samples were collected for bacteriological analysis from half of the randomly selected household water storage containers from both the intervention and the control villages at the baseline and the end of the study. Sterile 150 ml bottles were used for sample collection. The samples were transported to Haramaya University laboratory using ice packs and reached to the laboratory within 6 hours of collection. Multiple tube fermentation technique was used for determination of Escherichia coli which are regarded as the most reliable indicators of fecal contamination [6]. This technique is one of the standard methods in microbiological drinking water quality analysis [25].

### Statistical Analysis

Data were double entered on to EPI data Version 3.1 and statistical analysis was performed using STATA Version 11. Mean was calculated to determine average compliance with the intervention. Intention- to- treat analysis was used to compare the incidence of diarrhea among children under-five years of age between intervention and control arms. We presented incidence data because is a better predictor of disease transmission, disease surveillance and control [24]. Generalized estimation equation (GEE) with log link Poisson distribution family was used to consider the repeated and clustered nature of the data. Crude and adjusted incidence rate ratio along with corresponding 95% confidence intervals was calculated to control the potential confounders. We used mixed effect logistic regression to obtain intera-cluster correlation coefficient (ρ).

## Results

### Participants and Baseline Characteristics

A total of 427 children in the intervention and 422 children in the control arm enrolled in 36 clusters. The number of children per cluster was 24. The median number of participating households with children under-five years of age per cluster was 16. Three households refused to give consent. Four households were lost to follow up. Data obtained from 425 children (284households) in the intervention group and 420 children (285 households) in the control group. The Follow up started in June 2011 and ended in October 2011. Data was collected on the occurrence of diarrhea for 13499 person week observation representing 98.03% and 97% of the total person week observation in intervention and controls groups respectively (figure 1). There was no any harm observed in this study.

The mean ages of the respondents and that of the children under the age of five years were 29.06 years and 26 months respectively. Only 8% mothers/caregivers had formal education. There was no significant difference in baseline demographic and socio-economic characteristics between the intervention and the control households.

Some of the households were getting water from well (49.2%), some from spring (40.6%) and the rest from stream (10.19%). Almost all households store their drinking water using jerry-cans. Most of the households did not treat their drinking water. There was no significant difference on source of water and water treatment practice between the two groups at the baseline. About 37.6% of the households had latrine. Before the intervention, the two week prevalence of diarrhea was 24.3% in the intervention households and 25.2% in the control households. Overall, at the baseline, the intervention and the control households had similar sanitation, hygiene and water handling practices. The two groups were similar in many potential confounding variables, as well (Table 1).

**Table 1:** Baseline characteristics of community and household of the randomized control trial, Kersa district, Eastern Ethiopia, 2011.

Variable	Control	Intervention	P value
Number of clusters (neighborhoods)	18	18	
Number of households	285	284	
Number of under-five children	420	425	
Mean family size per household	5.79	6.26	0.15
Mean age of the children	26.16	26.56	0.65
Primary caregiver of children			
Mean age	29.6	29.3	0.45
No formal education	264 (92.6)	260(91.5)	0.63
Occupation (housewives)	276(96.8)	282(99.3)	0.31
Main occupation of the head of the household as farmer	278 (97.5)	273(96.1)	0.40
Economic indicators			
Own land	275(96.5)	279(98.2)	0.19
Own watch	119(41.8)	123(43.3)	0.70
Own mobile	27(9.5)	37(13)	0.18
Own television	10(3.5)	13(4.6)	0.51
Own radio	86(30.2)	90(31.7)	0.69
Primary water source			
Well	142(49.8)	138(48.6)	0.76
Spring	118(41.4)	113(39.8)	0.69
Stream/river	25(8.8)	33(11.6)	0.26
Domestic water treatment and storage			
Treat water before drinking (any method)	3(1)	5(1.7)	0.47
Storage water at home	285(100)	284(100)	NA*
Use Jerry can to store water	285(100)	283(99.6)	0.31
Sanitation and hygiene			
Place to wash hand	72(25.3)	68(23.9)	0.71
Soap available	36(12.6)	27(9.5)	0.23
Waste disposal (proper)	79 (27.7)	71(25)	0.48
Latrine present	106(37.1)	108(38)	0.83
Two week prevalence of diarrhea	106(25.2)	103(24.3)	0.73

* NA = not applicable.

### Diarrhea Incidence

**Figure 2:**
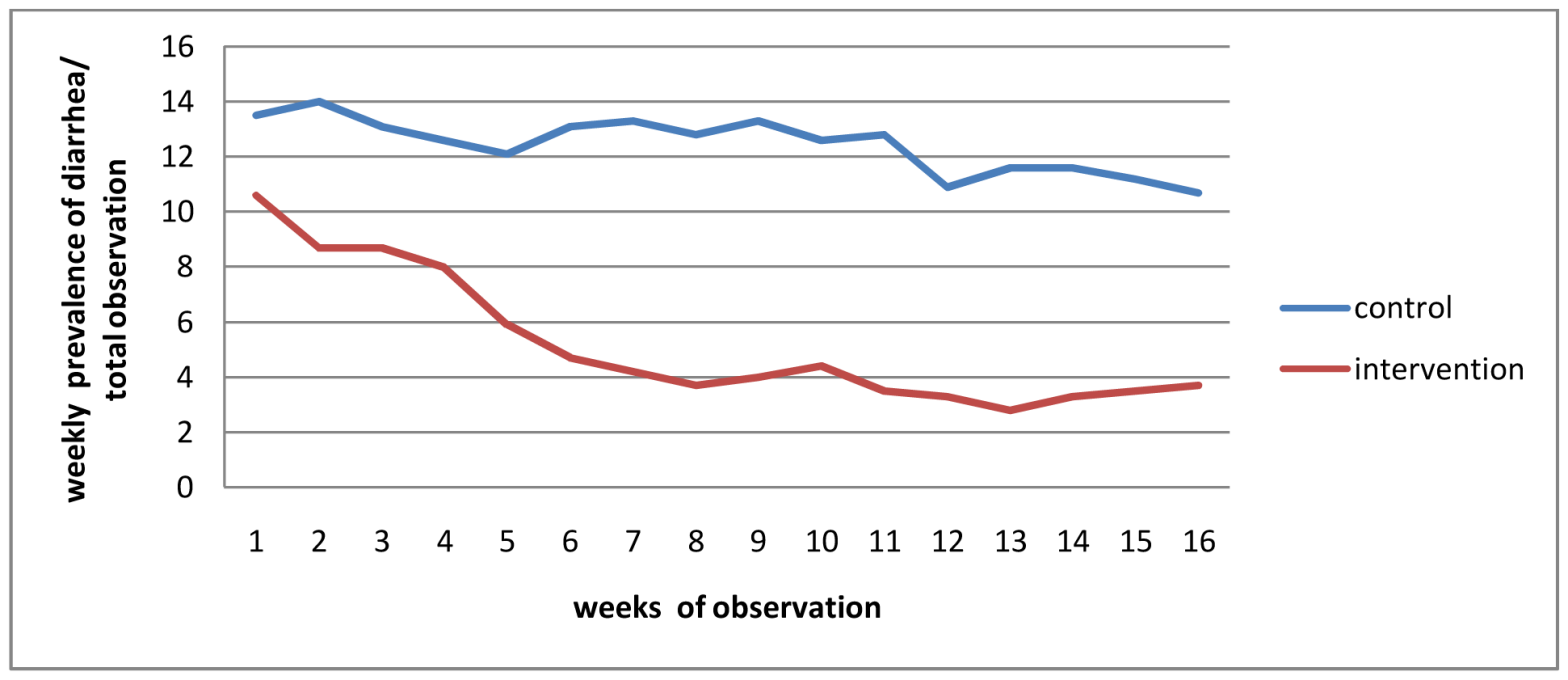
Weekly prevalence of diarrhea versus weeks of observation, Kersa district, Eastern Ethiopia, 2011.

From the control arm, 700 episodes of diarrhea (10.4 episodes per 100 person weeks observation) was reported but, from the intervention arm, it was 307 episodes (4.5 episodes per 100 person weeks observation). The effect of the intervention was different among the children with different age groups. It reduced 63% and 53% of the burden among children 3 to 4 years and 1 to 2 years of age respectively, while the reduction was lesser in less than one year children (44%) (Table 2). The relationship between study weeks and diarrhea in the intervention and control groups is shown in figure 2.

**Table 2:** Effect of the intervention with different age group of under-five children, Kersa district, Eastern Ethiopia, 2011.

Age group	Control groups (N = 420)			Intervention groups (N = 425)			% reduction in	P value
	Number ofDD episode	PWO	DD incidence	Number ofDD episode	PWO	DD incidence	DD incidence	
<1 year	104	1055	9.8	53	957	5.5	44	0.001
1–2 years	274	2173	12.6	131	2218	5.9	53	<0.001
3–4 years	322	3486	9.2	123	3610	3.4	63	<0.001
All <5 years	700	6714	10.4	307	6785	4.5	57	<0.001

DD = diarrhea diseases, PWO = Person week of observation. The incidence of diarrhea was calculated as the number of new episodes divided by the total number of person–weeks observation.

On the multivariable analysis adjusted for age, sex of the child, availability of latrine, waste disposal, availability of soap at home and hand washing facility, children in the intervention arm had lower risk of diarrhea (Adjusted RR = 0.42; 95% CI 0.37–0.49). There was a 58% overall reduction in the incidence of diarrhea among the intervention group compared to the controls (Table 3).

**Table 3:** Multivariable analysis of intervention effect on the incidence of diarrhea among under-five children, Kersa district, Eastern Ethiopia, 2011.

Factors	Crude IRR(95% CI)	Adjusted IRR(95% CI)	P value
Intervention	0.43(0.37–0.50)	0.42(0.36–0.48)	<0.001
Control	1	1	
Age of the child	0.88(0.83–0.94)	0.89(0.84–0.94)	<0.001
Sex of the child			
Female	0.98(0.85–1.14)	1.02(0.90–1.16)	0.709
Male	1	1	
Latrine available			
Yes	0.88(0.75–1.02)	0.99(0.85–1.15)	0.944
No	1	1	
Proper waste disposal			
Yes	0.82(0.69–0.97)	0.81(0.68–0.97)	0.027
No	1	1	
Soap available at home			
Yes	0.83(0.64–1.07)	0.88(0.68–1.14)	0.363
No	1	1	
Hand washing facility available			
Yes	0.93(0.78–1.10)	0.90(0.77–1.05)	0.218
No	1	1	

### Use of the Intervention

**Figure 3:**
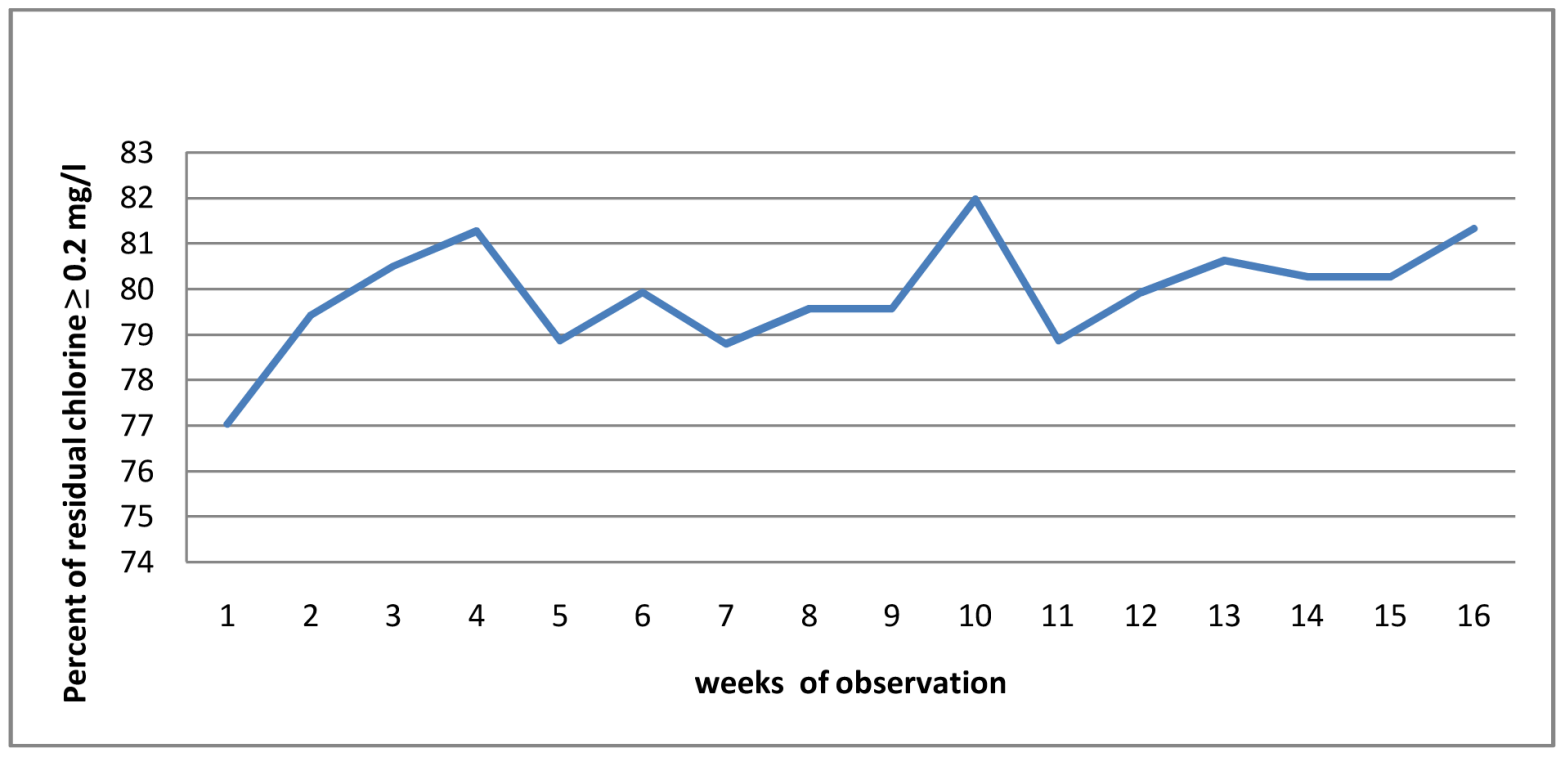
Percentage of residual chlorine during observation period, Kersa district, Eastern Ethiopia, 2011.

Field workers measured the residual chlorine on a weekly basis throughout the study. During a weekly scheduled visit, an average 79.89% of the samples from the intervention households had free residual chlorine ≥0.2 mg/l (Figure 3). In unannounced visit 75.94% and 77.13% of the households had free residual chlorine in the 5^th^ and 12^th^ week respectively. Members of the households were not informed about the result of the free residual chlorine test. During the follow up 1.25% of the control households reported treating their drinking water.

### Microbiological Quality

At the beginning and the end of the study drinking water samples were taken from the intervention and control household water storage containers. At the baseline, 78% of the households from the intervention (median E.coli MPN 70 per 100 ml) and 81% of those from the control households (median E.coli MPN 90 per 100 ml) were contaminated with E.coli. At the end of the study, however, 16.5% of the intervention households (median E.coli MPN 0 per 100 ml) and 65% control households (median E.coli MPN 60 per 100 ml) had E.coli in the sampled water. At the baseline, there was no significance difference between the two groups. However, drinking water samples from the intervention households were more likely to meet the WHO guidelines for bacteriological quality than samples from the control households at the end of the study (P<0.001) (Table 4).

**Table 4:** Household stored water quality among intervention and control households at the baseline and end point of intervention.

	Intervention households	Control households	P value
Number (%) of households with E.coli			
Baseline	109(78%)	114(81%)	0.53
End point	23(16.5%)	92(65%)	<0.001
Median E.coli per 100 ml of drinking water			
Baseline	70(0–1600)	90(0–900)	0.735
End point	0(0–280)	60(0–500)	<0.001

Median E.coli between the intervention and control households was compared using Wilcoxon rank sum test. The number of households with E.coli contamination between the two arms was compared using t-test.

## Discussion

A community based cluster randomized trial was conducted to assess the effectiveness of household water treatment with chlorine in reducing diarrhea among children under-five years of age. Household water treatment with sodium hypochlorite has reduced the incidence of diarrhea among children under-five years of age assigned to the intervention compared to the control (RR = 0.42 95% CI 0.37–0.49). There was significant improvement in the quality of stored water in intervention households.

Our result on reduction of diarrhea episode was consistent with similar studies conducted in Kenya [26] and Haiti [14]. But, the finding was higher than other studies from Bolivia [9] and Brazil [16]. The high magnitude of the protective effect in this study could be attributed to the high compliance of the intervention as observed during both scheduled and unannounced visits. Other study indicated that beneficial effect of such interventions may be greater in population where fecal contamination of drinking water is more likely [27]. We observed high fecal contamination of household stored water during the baseline thus that could be the reason for observing the desired effects of the intervention.

The household water treatment in this study was less effective among the younger children (less than one year) and the result is consistent with a study conducted in rural Guatemala [28]. This might be due to the high susceptibility and more chance of exposure to contaminated supplemental liquids among younger children.

High level of intervention compliance was achieved in the current study as observed in Zambia [10] and Gana [13]. Sub-group analysis within the intervention arm showed association between increased compliance and lower incidence of diarrhea (P = 0.014). Thus, the observed reduction in diarrheal episodes among the treatment group can be attributed to the intervention.

Household water chlorination is the most cost effective among all water quality interventions [29]. For instance, in the study area, 125 ml 1.25% sodium hypochlorite was bought $0.28 per bottle from retail shops or drug vendors, which can serve an average family for a month. This is about $ 0.00035 per liter that could be affordable for many of the households if they have access to the service. Using local community for the distribution of the disinfectant is a promising strategy to reach the rural community where the risk of diarrhea and other water borne diseases is high [30].

The primary outcome for this study was self reported diarrhea, which could be sensitive to recall bias. To minimize this recall bias, the data collection was on a weekly basis. This study was not blinded due to taste and odor of chlorine as well as ethical issue. Courtesy bias and Howthrone effect may overstate effectiveness of the HWT intervention in unblinded trials [2]. However, in this study, independent field workers were used for introducing the intervention and for collecting of data. Mausezahl et al recently indicated that in the absence of blinding using independent data collectors and intervention implementers is critical to reduce bias in HWT studies [31]. The investigators were not involved in the implementation of the intervention and data collection. The objective of the study was not stated for data collectors. We believe that sources of bias are addressed and their effects, if any, on the reported estimates are minimal.

In conclusion, home treatment of water with sodium hypochlorite significantly reduced the incidence of diarrhea among under-five children in the rural population where fecal contamination was high. We suggest that increasing access to such intervention would decrease child morbidity and mortality caused by diarrhea and help to achieve millennium development goal of 4 and 7.

## Supporting Information

Protocol S1 Trial Protocol.
(DOC)

Checklist S1 CONSORT Checklist.
(DOC)

## References

[1] WHO/UNICEF (2009) Diarrhoea: Why children are still dying and what can be done. Geneva/Newyork: The United Nations Children’s Fund/World Health Organization.

[2] Clasen T, Roberts I, Rabie T, Schmidt W, Cairncross S (2006) Interventions to improve water quality for preventing diarrhoea. Cochrane Database Syst Rev 3: CD004794. 10.1002/14651858.CD004794.pub2 16856059

[3] UNICEF/WHO (2012) Progress on Drinking Water and Sanitation: 2012 Update. Geneva,Swizerland: The United Nations Children’s Fund/World Health Organization. Available: www.wssinfo.org.

[4] WHO (2012) Rapid assessment of drinking-water quality: a handbook for implementation. Geneva, Swizerland: World Health Organization.

[5] WHO (2007) Combating waterborne disease at the household level. Geneva, Swizerland: World Health Organization.

[6] Wright J, Gundry S, Conroy R (2004) Household drinking water in developing countries: a systematic review of microbiological contamination between source and point-of-use. Tropical Medicine and International Health 9 (1): 106–117. 10.1046/j.1365-3156.2003.01160.x 14728614

[7] Brown J, Clasen T (2012) High Adherence Is Necessary to Realize Health Gains from Water Quality Interventions. PLoS ONE 7(5): e36735 doi:10.1371/journal.pone.0036735 22586491 10.1371/journal.pone.0036735 PMC3346738

[8] Semenza JC, Roberts L, Henderson A, Bogan J, Rubin CH (1998) Water distribution system and diarrheal disease transmission: a case study in Uzbekistan. The American Journal of Tropical Medicine and Hygiene 59(6): 941–946. 9886204 10.4269/ajtmh.1998.59.941

[9] Quick RE, Venczel LV, Mintz ED, Soleto L, Aparicio J, et al (1999) Diarrhoea prevention in Bolivia through point-of-use water treatment and safe storage: a promising new strategy. Epidemiol and Infection 122(1): 83–90. 10.1017/s0950268898001782 PMC2809591 10098789

[10] Quick RE, Kimura A, Thevos A, Tembo M, Shamputa I, et al (2002) Diarrhea prevention through household-level water disinfection and safe storage in Zambia. Am J Trop Med Hyg 66(5): 584–589. 12201595 10.4269/ajtmh.2002.66.584

[11] Reller ME, Mendoza CE, Lopez M (2003) A randomized controlled trial of household based flocculant-disinfectant drinking water for diarrhoea prevention in rural Guatemala. The American Journal of Tropical Medicine and Hygiene 69(4): 411–419. 14640502

[12] Luby SP, Agboatwalla M, Painter J, Altaf A, Billhimer W (2006) Combining drinking water treatment and hand washing for diarrhea prevention, a cluster randomised controlled trial. Tropical Medicine and International Health 11(4): 479–489. 16553931 10.1111/j.1365-3156.2006.01592.x

[13] Jain S, Sahanoon OK, Blanton E, Schmitz A, Wannemuehler KA, et al (2010) Sodium Dichloroisocyanurate Tablets for Routine Treatment of Household Drinking Water in Periurban Ghana: A Randomized Controlled Trial. Amrican Journal of Tropical Medicine and Hygiene 82(1): 16–22. 10.4269/ajtmh.2010.08-0584 PMC2803503 20064989

[14] Harshfield E, Lantagne D, Turbes A, Null C (2012) Evaluating the Sustained Health Impact of Household Chlorination of Drinking Water in Rural Haiti. Am J Trop Med Hyg 87(5): 786–795. 22987657 10.4269/ajtmh.2012.12-0010 PMC3516252

[15] Doocy S, Burnham G (2006) Point-of-use water treatment and diarrhoea reduction in the emergency context: an effectiveness trial in Liberia. Tropical Medicine and International Health 11(10): 1542–1552. 17002728 10.1111/j.1365-3156.2006.01704.x

[16] Kirchhoff LV, McClelland KE, Do Carmo Pinho M, Araujo JG, De Sousa MA, et al (1985) Feasibility and efficacy of in-home water chlorination in rural North-eastern Brazil. Journal of hygiene 94(2): 173–180. 2985691 10.1017/s0022172400061374 PMC2129411

[17] Schmidt WP, Cairncross S (2009) Household water treatment in poor populations: is there enough evidence for scaling up now? Environ Science and Technology 43(4): 986–992. 10.1021/es802232w 19320147

[18] Rosa G, Clasen T (2010) The scope of household water treatment in low- and medium-income countries. Am J Trop Med Hyg 82(2): 289–300. 20134007 10.4269/ajtmh.2010.09-0382 PMC2813171

[19] Hayes RJ, Bennett S (1999) Simple sample size calculation for clusterrandomized trials. International Journal of Epidemiology 28: 319–332. 10342698 10.1093/ije/28.2.319

[20] Mengistie B, Berhane Y, Worku A (2012) Prevalence of diarrhea and associated risk factors among under-five childern in Kersa distrct, Eastern Ethiopia. Article in presss.

[21] CDC (2008) Safe water for the community. A guide for establishing community based safe water system programme. Atlanta, USA: Center for Disease Control.

[22] Morris SS, Cousens SN, Lanata CF, Kirkwood BR (1994) Diarrhoea-defining the episode. Int J Epidemiology 23: 617–623. 10.1093/ije/23.3.617 7960391

[23] Wright JA, Gundry SW, Conroy R, Wood D, Du PM (2006) Defining episodes of diarrhoea: results from a three-country study in Sub-Saharan Africa. J Health Popul Nutr 24: 8–16. 16796145

[24] Schmidt WF, Arnold BF, Boisson S, Genser B, Luby SP, et al (2011) Epidemiological methods in diarrhoea studies – an update. International Journal of Epidemiology 40: 1678–1692. 22268237 10.1093/ije/dyr152 PMC3235024

[25] UNEP/WHO (1996) water quality monitoring: A practical guide to the design and implemntation of fresh water quality studies and monitoring programmes. London, UK: United Nation Environmental Programme/World Health Organization.

[26] Garrett P, Ogutu P, Mabonga P, Ombeki S, Mwaki A, et al (2008) Diarrhoea prevention in a high-risk rural Kenyan population through point-of-use chlorination, safe water storage, sanitation, and rainwater harvesting. Epidemiol and Infection 136: 1463–1471. 10.1017/S095026880700026X PMC2870746 18205977

[27] Lule JR, Mermin J, Ekwaru JP, Malamba S, Downing R, et al (2005) Effect of home-based water chlorination and safe storage on diarrhea among persons with human immunodeficiency virus in Uganda. Am J Trop Med Hyg 73(5): 926–933. 16282305

[28] Chiller TM, Mendoza EC, Lopez MB, Alvarez M, Hoekstra RM, et al (2006) Reducing diarrhoea in Guatemalan children: randomized controlled trial of flocculant-disinfectant for drinking-water. Bulletin of the World Health Organization 84(1): 28–35. 16501712 10.2471/blt.04.016980 PMC2626515

[29] Clasen T, Haller L, Walker D, Bartram J, Cairncross S (2007) Cost-effectiveness of water quality interventions for preventing diarrhoeal disease in developing countries Journal of Water and Health. 5(4): 599–608. 10.2166/wh.2007.010 17878570

[30] Clasen T (2008) Water Quality Interventions to Prevent Diarrhoea: Cost and Cost-Effectiveness. Geneva, Swizerland: World health organization.

[31] Mäusezahl D, Christen A, Duran Pacheco G, Tellez FA, Iriarte M, et al (2009) Solar Drinking Water Disinfection (SODIS) to Reduce Childhood Diarrhoea in Rural Bolivia: A Cluster-Randomized, Controlled Trial. PLoS Med 6(8): e1000125 doi:10.1371/journal.pmed.1000125 19688036 10.1371/journal.pmed.1000125 PMC2719054

