# Supplementary material for: Household Water Chlorination Reduces Incidence of Diarrhea among Under-Five Children in Rural Ethiopia: A Cluster Randomized Controlled Trial
Source: PLoS One. 2013 Oct 23;8(10):e77887. doi: 10.1371/journal.pone.0077887 (PMC3806844; doi:10.1371/journal.pone.0077887)
Supplement: Protocol S1 — Trial Protocol. (DOC) [file pone.0077887.s001.doc]

**Acceptability and effectiveness of household water treatment in reducing the prevalence of diarrhea among under-five children in Kersa District, Eastern Ethiopia**

**By**

**Bezatu Mengistie**

**Yemane Berhane**

**Alemayehu Worku**

**Randomized controlled trial study protocol**

**September 2011**

**Background**

Diarrhoea is the leading cause of morbidity and mortality, especially among children under five years of age in developing countries. The World Health Organisation (WHO) estimates that there are approximately 2.5 billion episodes and 1.5 million (nearly one in five) deaths annually in children under-five years of age which accounts for 21% of all deaths in developing countries. It kills more young children than Acquired Immunity Deficiency Syndrome (AIDS), malaria and measles combined. Africa and South Asia are home to more than 80 percent of child deaths due to diarrhoea (Black *et al*., 200; Parashar *et al*., 2003; UNICEF/WHO 2009).

Lack of access to safe drinking water is a major cause of diarrhoeal diseases. The Millennium development goal (MDGs) calls for reducing by half the proportion of people without sustainable access to safe drinking water. World health organization estimates that there are 1.1 billion people without access to safe drinking water. Progress towards this target is indicated by the proportion of households reporting the use of improved water source. However, research worldwide has shown that even drinking water which is safe at the source is subject to frequent and extensive faecal contamination during collection, storage and use in the home (Wright *et al*, 2004; James, 2005; Andrew, 2008; Othero *et al*., 2008).

To reduce this problem, point-of-use water treatment has been advocated as a means to substantially decrease the global burden of diarrhoea and to contribute to the MDGs. Point- of- use water treatment is the treatment of water at the household level to reduce the contamination of water at the source, during transport and storage. These include chlorination, filtration, solar disinfection, combined flocculation and disinfection, boiling and improved storage. Evidence on health outcome trials suggests that household water treatment (HWT) may reduce diarrhoea by 30-40%. However, There is large heterogeneity of effect estimates (Clasen *et al*., 2006; UNICEF/ WHO 2009).

Among the household water treatment options household-based chlorination is the most cost-effective where resources are limited, yielding high returns on every dollar invested mainly from lower health care costs but also from increased productivity and school attendance (Hutton, 2007; Clasen, 2007). It is promising approaches to supply safe water at the household level. Disinfection of water with chlorine and safe storage has resulted in significantly improving the microbial quality and reducing diarrheal disease (Semenza, 1998; Quick, 1999; Reller, 2003; Luby, 2004; Crump, 2005) The health impact gained from promoting point-of-use water treatment and safe storage varies considerably from one community to another and depends on a variety of technology-related as well as site-specific environmental and demographic factors. The gains for some communities may be very significant while for others may be relatively modest (Sobey, 2002; Thompson, 2003).

However, many researches are suggesting that much of the current apparent evidence for the effect of HWT may be strongly biased and that without other environmental improvements the benefits of HWT may be negligible (Kirchhoff, 1985; Eisenberg, 2000*;* Jain 2008; Schmidt, 2009). The limitations of current effect estimates due to responder and observer bias have been indicated by literatures (Arnold and Colford, 2007; Clasen *et al*., 2006; Fewtrell *et al*., 2005).

Based on the evidence it recommended the need for more research to identify the actual contribution that the intervention can make in preventing diarrhoea under different conditions, and not to overstate the effect due to bias. There are many unanswered questions about HWT especially with regard to effectiveness, acceptability in poor communities and identifying suitable target populations. Further development, implementation, evaluation and comparisons of household water treatment and safe storage technologies is both justified and encouraged (Thompson, 2003; Clasen, 2009; Schmidt, 2009).

Therefore this research will be conducted in to determine the acceptability and effectiveness of household water chlorination in reducing diarrhea episode and increasing weight gain among under-five children.

**Objective of the study**

**Primary objective:** To assess the effectiveness of household water chlorination in reducing the prevalence and incidence of diarrhea among under-five children

**Secondary objective:**

1. To determine the effectiveness of household water chlorination in increasing weight gain among under-five children.
2. To determine the acceptability of household water chlorination among the intervention households

**Study design and setting:** A community based randomised field trial will be conducted to determine the acceptability and effectiveness of household water disinfection with chlorine on reducing the prevalence and incidence of diarrhea and weight gain among under-five children in randomly selected clusters of Kersa Demographic and Health Research Centre field site. The study area has a population of 47,036 distributed among the 10 kebeles of which 7870 are children under-five years of age. All the households do not have running water. Families walk to collect water from springs, streams, or wells. The study will be conducted for a 16-weeks period from June to August 2011.

**Sample size**

The sample size is calculated using methods published by Hyes and Bennett (Hyes 1999). It is assumed that 11% incidence of diarrhea among children under-five years of age (baseline survey), 80% power, 10% drop out, 95% confidence interval and design effect of three from clustering. We calculated 18 clusters, 24 children under the age of five years per cluster will be followed for 16 weeks to detect 40% reduction in the incidence of diarrhea between the intervention and control groups.

**Enrolment and randomization**

We will use cluster level randomization to avoid ethical concerns and minimize the potential transfer of the intervention between the two groups. Ethiopian population census enumeration area map will be used to identify the clusters. There are 64 clusters (enumeration area) in study site. We will randomly select 36 clusters to participate in the study. Cluster selection will be done by central statistics authority using simple random sampling. Households in the selected clusters with at least one child under the age of five year will be identified by census. This is because we are expecting higher prevalence and incidence of diarrhea and the effect of the intervention would be higher among this group. Twenty four children will be selected randomly from the each cluster for follow up. Assigning of the clusters in to the intervention and control group will done by independent person using random sampling technique. Field workers will approach selected households in each cluster for baseline survey.

64 clusters

Randomly selected

36 cluster

**Randomization**

**Intervention Control**

18 clusters, 432 children

18 clusters, 432 children

**Follow up Follow up**

6921 person week observation

Intention to treat analysis

6921 person week observation

Intention to treat analysis

**An**

**Baseline survey:** we will collect basic information including demographic and socioeconomic information, water sources and water handling practices, access and water quality, housing conditions, and pre-intervention diarrhea rates.

**Interventions:** The intervention for this study will be 1.25% Sodium hypochlorite solution locally branded as “watergauard”. The intervention household will receive the hypochlorite disinfectant for household water treatment while the control groups will continue their usual practice. Both the intervention and control households use Jerricans for water collection and storage. Local women will be selected for the distribution of the intervention (sodium hypochlorite). They will be trained on how to use sodium hypochlorite for household water treatment and explain to the intervention households how to treat water. The intervention will be distributed for 16 weeks.

**Outcomes:**

Primary outcome for this study is the occurrence of diarrhea among under-five children. Diarrhoea is defined as three or more loose or watery stools in 24 hours. Diarrhea is considered as new episode if it occurred after a period of three diarrhea free days (Morris 1994, Wright 2006). Incidence will be calculated as the number of new episodes divided by the total number of person–weeks observation and longitudinal prevalence as the number of weeks of diarrhea divided by the total number of weeks of observation for each child ( Morris 1996, Schmidt 2011) .

**Inclusion and exclusion criteria**

Inclusion criteria: Children under the age of five years will be included in the study.

Exclusion criteria: children who are seriously sick with other disease at the time of the study.

**Diarrhoea monitoring**

The survey instrument will be pre-tested and validated in the nearby villages. Field workers will use a validated questionnaire to record the occurrence of diarrhea in children under the age of five years, water treatment practices and residual chlorine in the selected households on weekly bases. A female caregiver will provide the information.

**Anthropometric measurement:** The secondary outcome for this study is weight gain. All fieldworkers will be trained on anthropometric measurement techniques over two days of training, and they will collect measurements in teams of two. Fieldworkers will measure the weight of children using digital scales. These scales will be calibrated every day before starting the data collection. Anthropometric measurement will be taken at the baseline and the end of the

Study.

**Laboratory measurements**

Residual free chlorine concentration will be measured from the samples of stored household water once a week and on unannounced visits by using the residual chlorine test kit (Wagetech 225 comparator colour disc**) .**Water samples will be collected from randomly selected 50% of the households water storage containers from both the intervention and control groups at the baseline and end of the study. The samples will be collected with sterile technique in 150 ml sterile plastic vessels containing sodium thiosulfate to neutralise any free chlorine in the water samples. Environmental health science graduates will collect samples and transport with icepacks to the Haramaya University bacteriology laboratory for processing within six hours of collection for analysis**.** Most probable number (MPN) will be used for the analysis of the sample and MPN table to quantify Escherichia coli which are regarded as the most reliable indicator of fecal contamination (wright 2004)

**Statistical analysis**

All the data will be double entered into EPI info. Intention- to- treat analysis will be applied to compare between the intervention and control groups. Mean will be calculated to determine the proportion of households who use the intervention. Generalized Estimation Equation (GEE) will be used to compare the prevalence and incidence of diarrhea between the intervention and control arms while T-test will be used to compare the mean weight gain between the two groups.

**Ethics**

The study reviewed and approved by the Haramaya University, College of Health Science ethical review committee. The purpose of the study will be explained to the participants. Written consent will be obtained from the caregivers of children. They will be given enough time to decide to participate or to say no. If they do not wish to answer any questions during the data collection, they can say no and the interviewer will move on to the next questions. They will be informed to ask questions at any time. Oral consent will be secured from the partner of the caregiver, community leaders and local administrators. The researcher will arrange the provision of ORS to mothers/caregivers whose children are with diarrhoea at the time of study and advise to attend the nearby health institution.

**Risks and discomforts**

There are no known risks related to the study.

**Benefits**

The intervention may reduce the prevalence and incidence of diarrhoea and other water related diseases. Therefore, the family as a whole could directly benefited from the intervention. The benefit is expected to be more in under-five children where the prevalence of diarrhoea is high.

**Confidentiality**

We will not be sharing the information out side of the research team. The information that we collect from this research project will be kept confidential. Information collected from the research will be put away and no-one but the researchers will be able to see it. Any information will have a number on it instead of his /her name. It will not be shared with or given to anyone except the investigator or data clerk.

**Sharing of research findings**

At the end of the study, we will be sharing what we have learnt with the participants and with the community. We will do this by meeting first with the participants and then with the larger community. A written report will also be given to the participants whom they can share with their families. We will also publish the results in order that other interested people may learn from our research.

**Limitation and the strength of the study**

The study is not free from limitations. It is not possible to make this study blinded due to the nature of the intervention, but we will assign separate group of intervention implementers and data collectors to reduce observer responder bias.

References

Black, R.E., Morris, S.S. and Bryce, J., 2003. Where and why are 10 million children dying every year? Lancet. 361: 2226-34.

Parashar UD., Hummelman E.G., Bresse, J.S. and Glass, I.R., 2003. “Global Illness and Deaths caused by Rotavirus Disease in Children.” Emerging Infectious Diseases. 9: 565-72

UNICEF/WHO., 2009. Diarrhoea: Why children are still dying and what can be done. The United Nations Children’s Fund (UNICEF)/World Health Organization (WHO).

Wright, J., Gundry, S. and Conroy, R., 2004. Household drinking water in developing countries: a systematic review of microbiological contamination between source and point-of-use. Tropical Medicine and International Health. 9 (1): 1

James, K.T., 2005.Clean drinking water for homes in Africa and other less developed countries: flocculants-disinfectant treatment with bleach is effective and acceptable. BMJ. 331:468-9.

Andrew, F.T. and Richard CC., 2008. Targeting appropriate interventions to minimize deterioration of drinking water quality in developing countries. Health Population Nutrition. 26(2): 125-138.

Othero, D.m., Orago, A.S. and Groenewegen, T., 2008. Home management of diarrhoea among under-fives in a rural community in Kenya: Household perceptions and practices. East Afr J Public Health. 5(3): 142-6.

Clasen, T., Roberts, I., Rabie, T., Schmidt, W. and Cairncross, S., 2006. Interventions to improve water quality for preventing diarrhoea. Cochrane Database Syst. Rev. 3:CD004794.

Hutton 2007

Semenza, J.C., Roberts, L., Henderson, A., Bogan, J. and Rubin, C.H., 1998. Water distribution system and diarrheal disease transmission: a case study in Uzbekistan. The American Journal of Tropical Medicine and Hygiene. 59, 941–946.

Quick, R.E., 1999. Diarrhoea prevention in Bolivia through point-of-use water treatment and safe storage: a promising new strategy. Infect disease Epidemiology. 122(1): 83–90.

Luby, S.P., Mendoza, C., Keswick, B.H., Chiller, T.M. and Hoekstra, R.M., 2008. Difficulties in Bringing Point-of-Use Water Treatment to Scale in Rural Guatemala. Am. J. Trop. Med. Hyg. 78(3): 382–387

Crump JA, Otieno PO, Slutsker L, Keswick BH, Rosen DH, Hoekstra RM, Vulule JM, Luby SP, 2005. Household based treatment of drinking water with flocculant-disinfectant for preventing diarrhoea in areas with turbid source water in rural western Kenya: Cluster randomised controlled trial. *BMJ 331:*478.

Clasen, T., Haller, L., Walker,D., Batram, J.,and cairncrosis S., 2007. Cost-effectiveness of water quality interventions for preventing diarrhoeal disease in developing countries. Journal of Water and Health. 5(4):599-608.

Reller, M.E., Mendoza, C.E. and Lopez, M.B., 2003. A randomized controlled trial of household based flocculant–disinfectant drinking water for diarrhoea prevention in rural Guatemala. The American Journal of Tropical Medicine and Hygiene. 69: 411–419.

Luby 2004

Thompson, T., Sobsey, M.D. and Bartram, J., 2003. Providing clean water, keeping water clean: an integrated approach. International Journal of Environmental Health Research. 13:89-94.

Sobsey, M.D., Handzel, T. and Venczel, L., 2003. Chlorination and safe storage of household drinking water in developing countries to reduce waterborne disease. Water Science and Technology. 47(3): 221–228.

Kirchhoff, L.V., McClelland, K .E., Carmo, P. M., Araujo, J. G., Sousa, M. A. and Guerrant, R. L., 1985. Feasibility and efficacy of in-home water chlorination in rural North-eastern Brazil. J. Hyg. (Lond). 94 (2): 173–180.

Eisenberg, J.N., Scott, J.C. and Porco, T., 2007. Integrating disease control strategies: Balancing water sanitation and hygiene interventions to reduce diarrheal disease burden (plus letter and reply). Am. J. Public Health. 97 (5), 846–852.

Jain, S., Sahanoon, OK., Blanton, E., Schmitz, A., Wannemuehler, KA., 2010. Sodium Dichloroisocyanurate Tablets for Routine Treatment of Household Drinking Water in Periurban Ghana: A Randomized Controlled Trial. Am J Trop Med Hyg 82: 16-22.

Schmidt, W.P. and Cairncross, S., 2009. Household water treatment in poor populations: is there enough evidence for scaling up now? Environ Sci Technol. 15;43 (4):986-92.

Fewtrell, L., Kaufmann, R. B., Kay, D., Enanoria, W., Haller, L. and Colford, J. M., 2005. Water, sanitation, and hygiene interventions to reduce diarrhoea in less developed countries: A systematic review and meta-analysis. Lancet. Infect. Dis*.*  *5* (1), 42–52.

Arnold, BF., Colford, JM., 2007. Treating water with chlorine at point-of-use to improve water quality and reduce child diarrhea in developing countries: a systematic review and meta-analysis. Am J Trop Med Hyg 76: 354–364.

Hayes, RJ., Bennett, S., 1999. Sample size calculation for cluster randomized trials. International journal of Epidemiology 28: 319-332.

Morris, SS., Cousens, SN., Lanata, CF., Kirkwood, BR., 1994. Diarrhoea-defining the episode. International Journal of epidemiology 23: 617-623.

Wright, JA., Gundry, SW., Conroy, R., Wood, D., Du, PM., 2006. Defining episodes of diarrhea: result from a three-country study in Sub-Saharan Africa. Journal of health population and nutrtion 24: 8-16.

Morris, SS., Cousens, SN., Kirkwood, BR., Arthur, P., Ross, DA., 1996. Is prevalence of diarrhea a better predictor of subsequent mortality and weight gain than diarrhea incidence? Am J Epidemiol *144*

Schmidt, WF., Arnold, BF., Boisson, S., Genser, B., Luby, SP., et al. 2011. Epidemiological methods in diarrhea studies _ an update. Intrenational Journal of Epidemiology 40: 1678-1692.
